# Tumor‐stromal crosstalk and macrophage enrichment are associated with chemotherapy response in bladder cancer

**DOI:** 10.1002/2211-5463.70179

**Published:** 2025-12-12

**Authors:** Sophie Leypold, Janik Riese, Lancelot Seillier, Mark Kühnel, Julia Pannhausen, Charlotte O. J. Fröhlich, Christian Martin, Peter Boor, Matthias Saar, Danny D. Jonigk, Nadine T. Gaisa, Michael Rose

**Affiliations:** ^1^ Institute of Pathology, University Hospital RWTH Aachen University Germany; ^2^ Center for Integrated Oncology (CIO) Düsseldorf CIO Aachen Bonn Köln Düsseldorf Germany; ^3^ Institute of Pharmacology and Toxicology RWTH Aachen University Germany; ^4^ Department for Nephrology and Clinical Immunology RWTH Aachen University Hospital Germany; ^5^ Department of Urology and Pediatric Urology, University Hospital RWTH Aachen University Germany; ^6^ German Center for Lung Research, DZL, BREATH Hanover Germany; ^7^ Institute of Pathology, University Hospital University of Ulm Germany

**Keywords:** bladder cancer, chemokines, chemotherapy, M2 macrophages, tumor‐associated macrophage, tumor‐stroma crosstalk

## Abstract

Gemcitabine/Cisplatin (Gem/Cis) chemotherapy is a standard treatment for muscle‐invasive bladder cancer (MIBC) but yields suboptimal response rates. The contribution of tumor‐stromal crosstalk and macrophage recruitment to chemoresistance remains poorly understood. This study investigated these mechanisms using a functional *ex vivo* bladder cancer tissue slice model combined with *n* = 64 spatial transcriptomics. Spatial analysis revealed transcriptomic changes involving the immunomodulating gene *SPP1* that has been also recently presented as a putative predictive biomarker for neoadjuvant chemotherapy in bladder cancer. Moreover, Non‐Responders exhibited upregulation of chemokines including *CXCL1* and *CXCL8* and enrichment of immunoregulatory M2 macrophages in tumor regions, suggesting active macrophage recruitment from the stroma. On the contrary, Responders showed upregulation of complement components, proinflammatory macrophage subsets and signals associated with cytotoxic lymphocyte recruitment. Tissue slices corresponding cell cultures confirmed overexpression of immunomodulating markers including checkpoints *PD‐L1* and *PD‐L2* in Non‐Responder cancer cells upon Gem/Cis treatment. Using TCGA bladder cancer data, the transcriptomic gene set was further validated revealing a prognostic signature associated with patients' outcome. These findings uncover a novel mechanism of chemotherapy resistance in bladder cancer driven by tumor–stromal interactions and macrophage recruitment and suggest that targeting macrophage infiltration may improve chemotherapy response in bladder cancer.

## Graphical abstract

Chemoresistance in bladder cancer: Macrophage recruitment associated with CXCL1, CXCL5 and CXCL8 expression is characteristic of Gemcitabine/Cisplatin (Gem/Cis) Non‐Responder tumors (right side) while Responder tumors did not show substantial tumor‐stromal crosstalk (left side). All biological icons are attributed to Bioicons: carcinoma, cancerous‐cell‐1,2,3,4,5, (apoptotic adaptation) fibroblast‐1,2,3,4,5, dendritic‐cell‐2, macrophage, nk‐cell icon by Servier https://smart.servier.com/ is licensed under CC‐BY 3.0 Unported https://creativecommons.org/licenses/by/3.0/.

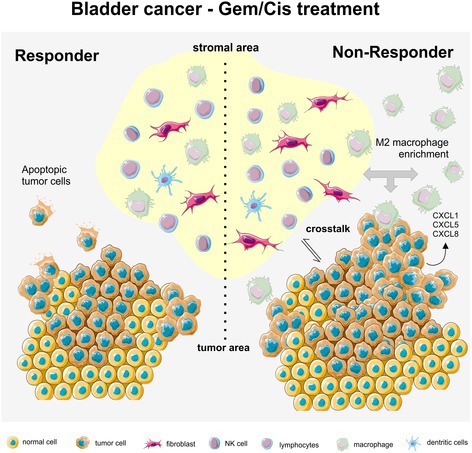

**Abbreviations**
CXCL: C‐X‐C motif chemokine ligand
FFPE: formalin‐fixed and paraffin embedded
Gem/Cis: gemcitabine/cisplatin
IC50: half maximal inhibitory concentration
MIBC: muscle‐invasive bladder cancer
MMP: matrix metalloproteinases
MSC: medium‐treated controls
ORR: overall response rate
ROI: region of interest
SCC: squamous cell carcinoma
TAM: tumor‐associated macrophage
UC: urothelial carcinoma

Bladder cancer is the 9th most common cancer with an estimated 613 791 new cases worldwide, and the 13th leading cause of cancer‐related mortality [1]. Approximately 70–75% of patients present with nonmuscle‐invasive bladder cancer (NMIBC), while 20–25% are diagnosed with muscle‐invasive bladder cancer (MIBC), and 5% already have metastatic disease at the time of diagnosis [2]. The 5‐year survival rate for regionally advanced bladder cancer, meaning the tumor has invaded nearby tissues or pelvic lymph nodes, is roughly 36%, whereas it drops to about 5% when distant metastases are present, defined as dissemination to remote organs or nonregional lymph nodes [3].

Radical cystectomy accompanied by (neo‐) adjuvant chemotherapy has been a standard of care for more than 30 years. As early as 1990, Loehrer *et al*. demonstrated the efficacy of cisplatin‐based combination chemotherapy in metastatic urothelial carcinoma (UC) [4]. Meanwhile, tremendous advances in understanding the tumor biology of bladder cancer have been made, and new therapeutic options such as immune checkpoint inhibitor (ICI)‐guided immunotherapy and antibody‐drug‐conjugates evolved. Nevertheless, cisplatin‐based combination chemotherapy continues to play a role in the treatment of muscle‐invasive bladder cancer, in the both neoadjuvant and adjuvant settings [5]. Although cisplatin‐based chemotherapy reduces bladder cancer‐related mortality, the initial overall response rate (ORR) in clinical practice ranges from 40 to 60% [6] while the majority of patients will progress or become refractory to treatment [7].

Over the past 10 years, intensive efforts have been made to therapeutic stratification of MIBC patients for chemoresponse. A good example is the attention paid to the transcriptomic profiling of MIBC allowing to classify these tumors into six distinct molecular subtypes [8] comprising different groups of luminal and basal/squamous cancers associated with heterogeneous outcomes for chemotherapy, targeted therapy, and ICI [9]. Beyond that accumulating studies aimed at identifying molecular biomarkers helping to predict chemotherapy response including the overexpression of CD147 and monocarboxylate transporter 1 (MCT1), as well as the upregulation of pyruvate kinase M2 (PKM2) and activation of OIP5 [10, 11, 12]. However, understanding intrinsic and acquired mechanisms of chemoresistance, which limit treatment efficacy, remained challenging since recent research mainly from other tumor entities proposed a substantial role of the stromal and immune microenvironment, especially tumor‐associated macrophage (TAM) recruitment. In esophageal cancer, for instance, Yamamoto *et al*. showed that tumor infiltrating M2 macrophage predicted response to chemotherapy [13]. Mechanistically, Yin *et al*. revealed a crosstalk between TAM and colorectal cancer cells via the IL6R/STAT3/miR‐204‐5p axis while in gastric cancer, the CXCL5/PI3K/AKT/mTOR pathway was proposed to trigger chemoresistance of tumor cells [14, 15]. In bladder cancer, a retrospective study by Wang *et al*. also suggests an impact of the tumor‐associated macrophages as higher clinical response rates were associated with low M2 macrophage and high T‐cell infiltration [16]. Interestingly, other studies mostly demonstrated poor prognosis associated with M2 macrophage enrichment [16, 17]. In 2022, the study by Sun *et al*. differed between TAM and the M2/M1 ratio in distinct bladder cancer patient groups using retrospective cohorts concluding that the infiltration and polarization status of TAMs might indeed provide tools to tailor chemotherapy but evidence derived from a more functional model is still missing [18, 19]. Hence, apart from that, the role of the tumor‐associated stroma and TAMs in chemotherapeutic response remains poorly understood. We aimed to give novel insights into a putative crosstalk between stromal and tumor components upon Gem/Cis treatment using precision tissue slices of bladder tissues *ex vivo* followed by spatial transcriptomic profiling.

## Methods

### Patient samples, tissue sectioning, primary cell cultures, cultivation, and treatment

A total of 15 muscle‐invasive bladder carcinomas were examined, including seven pure urothelial carcinomas, four urothelial carcinomas with divergent differentiation (squamous and small cell neuroendocrine), and four pure squamous cell carcinomas (Table S1). Tissue cores with a diameter of 10 mm were taken from native cystectomy specimens. From these cores, 500‐μm‐thick tissue slices were prepared using a Krumdieck tissue slicer (Alabama Research and Development, Munford, AL, USA). The slices were transferred into cell culture dishes and cultured in a specialized medium (Mammary Epithelial Cell Growth Basal Medium (MEBM) [20]). The application of Gemcitabine/Cisplatin (Gem/Cis) was based on mean IC50 values determined from cell culture experiments using various bladder cancer cell lines [20]. Accordingly, Gem/Cis was applied at concentrations of 5× and 10× IC50 (IC50 Gem/Cis: 0.25 μm/7.5 μm) and subsequently cultured for a maximum of 48 h at 37 °C and 5% CO_2_. The IC50 values were adjusted to previous calculations based on *in vitro* data from various bladder cancer cell lines, as previously published [20] (Table S2). Control tissues included both untreated cultured tissue slices and native, noncultured tissue slices. After 48 h, the samples were fixed in formalin for 12–24 h, followed by paraffin embedding. Corresponding tissue samples located close to the above‐described tissue cores were used for establishing patient‐derived *ex vivo* lines as described previously [20] reflecting a Responder and Non‐Responder cell line with long‐term growth features (passages > 10). Analogous to the tissue slices, the *ex vivo* cultured cancer cells were treated with Gem/Cis. In order to ensure stable and valid expression data, final concentrations of 0.1 μm (Gem) and 1.0 μm (Cis) were applied followed by RNA extraction 24 h and 72 h after Gem/Cis treatment. Experiments were in accordance with the Declaration of Helsinki and with the regulations of the Institutional Review Board (IRB)‐approved protocols of the Medical Faculty (EK 268/21, EK 206/09 study number 199, 304 and 311). Written patient consent was available through the RWTH Centralized Biomaterial Bank (RWTH cBMB).

### RNA extraction

RNA isolation from cultured cells was performed using the NucleoSpin^®^ RNA Plus Kit (740984.50; Macherey‐Nagel, Düren, Germany) according to the manufacturer's instructions.

### cDNA synthesis and quantitative real time reverse transcription PCR

For cDNA synthesis, the Reverse Transcription System Kit (A3500; Promega, Walldorf, Germany) with 2 μg total RNA was used on a Bio‐Rad C1000 Touch Thermal Cycler. Quantifying mRNA‐expression was done by real‐time qPCR (RT qPCR) on a CFX96 Touch system (Bio‐Rad, Hercules, CA, USA) using iQ™ SYBR^®^ Green Supermix (1 725 125; Bio‐Rad) and Power SYBR^TM^ Green PCR Master Mix (4 367 659; Thermo Fisher, Waltham, MA, USA). Samples were measured in triplicate. Relative expressions were calculated via the 2^−∆∆Ct^ method using *GAPDH* as reference gene. Primer sequences and PCR conditions are listed in Table S3.

### Preparation of paraffin sections and immunohistochemical staining

From the formalin‐fixed, paraffin‐embedded (FFPE) tissue samples, 3‐μm‐thick paraffin sections were prepared. These sections were then stained with hematoxylin–eosin (H&E) and antibodies against Ki67 (Clone: MIB‐1, DAKO, Santa Clara, CA, USA) and Cleaved Caspase‐3 (Clone: Asp175, Cell Signaling, Danvers, MA, USA) according to the manufacturer's instructions using a Dako Autostainer instrument (Dako AS, Glostrup, Denmark).

### GeoMx spatial transcriptomics

The Bruker GeoMx DSP platform (Bruker, Seattle, WA, USA) was used in conjunction with the whole transcriptome atlas (WTA). FFPE precision‐cut bladder slices, following the previously described treatment, were immobilized onto slides by drying at 60 °C for 3 h prior to the assay. Samples were deparaffinized through a descending solvent series: three 5‐min washes in xylene, followed by two 5‐min washes in 100% ethanol, one 5‐min wash in 95% ethanol, and a 1‐min rinse in 1× PBS. The slides were then incubated in 1× Tris‐EDTA (pH 9.0) in a pressure cooker at low pressure for 20 min at 99 °C for antigen retrieval. Proteinase K treatment in PBS was applied at 37 °C for 15 min to further retrieve the targets. After washing thoroughly with PBS, samples were postfixed in 10% neutral buffered formalin (NBF) for 5 min, followed by two 5‐min washes in NBF stop buffer and a 5‐min wash in PBS. Next, GeoMx RNA probes were hybridized overnight (16–17 h) at 37 °C in a ZytoBrite Hybridizer. PBS was replaced with 200 μL of hybridization solution containing the probe mix (WTA Whole Transcriptome Atlas Human RNA, Bruker) per slide, which was then covered with a HybriSlip (Grace Bio‐lab, Sigma Aldrich, Saint Louis, MO, USA). After incubation, unbound probes were removed by washing in a prewarmed solution containing 50% formamide and 2× SSC. Coverslips were removed by submerging the slides in washing solution, followed by two 25‐min washes at 37 °C and two 2‐min washes in 2× SSC at room temperature. Tumor tissue was detected via Cytokeratin staining (Fig. S1). Slides were blocked with buffer W for 30 min in a humidified, light‐protected chamber. The blocking buffer was replaced with 200 μL of an antibody mix, which was incubated for 1 h at room temperature in the dark. The anti‐Cytokeratin, fluorescence‐labeled antibody (Novus Biologicals, NBP2‐33200AF488, Littleton, CO, USA) was used at a 1 : 500 dilution, along with SYTO83 (Invitrogen, S11364, Carlsbad, CA, USA) at a 1 : 25000 dilution. After incubation, the slides were washed and stored in 2× SSC. The slides were then washed twice for 5 min in 2× SSC before being placed in the GeoMx DSP slide holder with 3 mL of buffer S. The slides were scanned on the GeoMx DSP platform, with exposures of 200 ms for Cytokeratin and 50 ms for SYTO83, using 488 nm and 559 nm illumination. Regions of interest (ROIs) were defined, and oligonucleotides were photo‐released. The DSP samples were then collected in a 96‐well PCR plate for downstream processing. The 96‐well plates were sealed with air‐permeable foil and allowed to dry completely overnight under a hood. Next, the DSP samples were rehydrated with 10 μL of DEPC‐treated water, covered with an adhesive seal, and incubated at room temperature for 10–20 min. Library preparation was performed in a new 96‐well PCR plate, using 2 μL of GeoMx NGS Master Mix, 4 μL of Seq Code Primer Mix (from the corresponding 96‐well plate), and 4 μL of DSP sample from the collection plate using a MiniAmp Thermal Cycler (Applied Biosystems) with a 100 °C heated lid. After PCR amplification, all samples, including internal no‐template controls (NTC), were collected into a single 1.5‐mL tube and purified using magnetic beads under nuclease‐free conditions. The library quality was assessed via TapeStation analysis, with the characteristic peak at 170 bp. Next‐generation sequencing (NGS) was performed using the NovaSeq 6000 platform, with 250 pM of library and 5% spike‐in as an internal control.

### Data processing

FASTQ files were initially processed using the GeoMx NGS Pipeline (Bruker) to convert raw sequencing data into a usable format for analysis. Following this, quality control (QC) measures were applied to ensure data integrity and reliability. The primary QC thresholds included a minimum of 1000 raw reads per region of interest (ROI), a sequencing saturation rate greater than 50%, and a minimum of 80% of the reads being aligned, stitched, and trimmed. Additionally, ROIs that exhibited expression of fewer than 10% of the genes were excluded from further analysis to ensure the inclusion of only biologically meaningful data. After applying these QC criteria, three ROIs were excluded due to insufficient quality, leaving a total of 63 ROIs representing tumor regions across eight different donors, as well as 64 ROIs corresponding to adjacent stroma. To further refine the dataset, genes that were undetected in 10% or more of the ROIs were removed. This process reduced the gene list to 18 677 genes. Finally, raw count data underwent negative RNA probe‐based quantile normalization (Q3 normalization).

### *In silico* validation using TCGA data

For external validation of the Non‐Responder transcriptomic signature, RNA‐seq expression data from The Cancer Genome Atlas (TCGA) were analyzed. TCGA bladder cancer (BLCA) cohort data were accessed through the Genomic Data Commons (GDC) portal (release version 32.0) using the TCGAbiolinks R package (v2.28.3) in R (v4.3.2). Clinical treatment annotations were reviewed, and 61 patients treated with platinum‐based (cisplatin or carboplatin) or gemcitabine‐containing chemotherapy were identified for inclusion in the analysis. Gene‐level expression values (HTSeq‐FPKM‐UQ normalized) were retrieved for all transcripts previously defined as part of the Non‐Responder signature. Unsupervised hierarchical clustering of the selected transcripts was performed using Euclidean distance and complete linkage implemented in the pheatmap package (v1.0.12). Associations between gene expression and overall survival were evaluated by univariable Cox proportional hazards regression using the survival package (v3.5). Hazard ratios (HR) and 95% confidence intervals were calculated for each gene. Kaplan–Meier survival analyses were conducted by stratifying patients according to median expression of the composite signature, and statistical significance was assessed with the log‐rank test. To investigate functional relevance, pathway enrichment analysis was performed on Non‐Responder signature genes with significant HR (*P* < 0.05) using the clusterProfiler R package (v4.8.1). The Gene Ontology (GO) Biological Process 2025 database was used as the reference, applying Benjamini–Hochberg correction for multiple testing. Enriched processes included cell migration, stromal interaction, angiogenesis and extracellular matrix remodeling. The datasets analyzed during this study are publicly available from The Cancer Genome Atlas (TCGA) through the Genomic Data Commons (GDC) data portal (https://portal.gdc.cancer.gov/).

### Statistics

Statistical analysis was carried out using R Studio (R version 4.0.1) and Jupyter (Python version 3.12.7) [21]. Each ROI was treated as an independent replicate and an adjusted *P*‐value threshold of 0.05 was used for all statistical tests. The python package pydeseq2 was used to calculate a linear mixed model of expression levels with correction for histological entity was used to identify differentially expressed transcripts [22]. Gene set enrichment and upstream activator analysis of differentially expressed genes was performed using QIAGEN IPA [23] (QIAGEN Inc.). Cellular deconvolution of common immune cell populations was performed using the xCell algorithm [24]. Enrichment analysis of TAM subtypes was performed as previously described [25, 26]. Active ligand signaling events were predicted by examining the Pearson's correlation between ligands and their target genes between sender and receiver regions using the squidpy python package [27]. All data required to replicate the analysis carried out in this paper is presented in the Supporting Information (Gene Expression Omnibus). This includes raw and normalized, ROI annotations and differentially expressed gene results for ROI regions [28].

## Results

### Classifying bladder cancer tissues into chemotherapy Responders and Non‐Responders

**Fig. 1:**
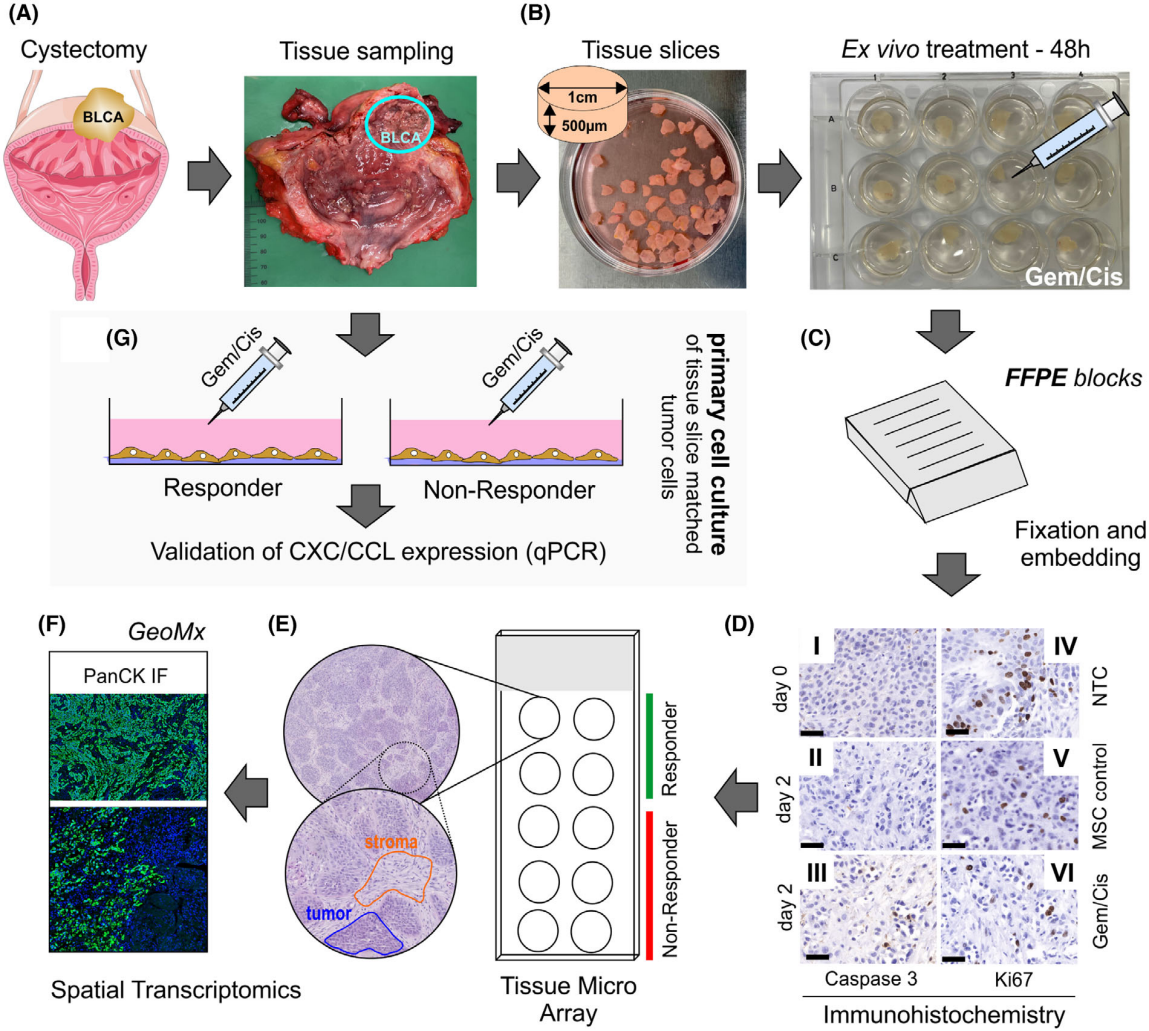
Study workflow. (A, B) Bladder cancer tissues derived from cystectomies were harvested and precisely cut using an Alabama R&D Tissue Slicer into 500 μm slices (*n* = 30 to 50), cultured for 48 h in specialized cell medium (MSC) in 12‐well plates at 37 °C and treated with Gemcitabine and Cisplatin (Gem/Cis) in two different concentrations (5×IC50 (1,25 μm/37,5 μm) and 10×IC50 (2,5 μm/75 μm)). Additional MSC medium was used as control to normalize all drug‐associated values. (C, D) After drug treatment the formalin‐fixed and paraffin embedded (FFPE) tissue slices were used for immunohistochemical staining of an apoptosis (Caspase 3) and proliferation (Ki67) marker to classify the cancers into two groups: Responder (cutoff marker expression: 200% compared to the control) and Non‐Responder (cutoff marker expression: < 200% compared to the control). A non‐treated control (NTC) served as reference tissue to compare immunohistochemical markers on day 0 to assess putative influences of *ex vivo* culture conditions. (E) A tissue micro array was then prepared and (F) used for spatial (whole) transcriptomic analyses based on the GeoMx (Nanostring) platform. Overall *n* = 32 stromal and *n* = 32 tumor areas were profiled, and results were analyzed by using the Ingenuity Pathway software (Qiagen). (G) Corresponding tissue samples that were located close to the tissue cores were further used for establishing patient‐derived *ex vivo* lines reflecting a Responder and Non‐Responder cell line with long‐term growth features (passages > 10). Analogously to the tissue slices, the *in vitro* cultured cancer cells were treated with Gem/Cis and mRNA expression of genes encoding chemokines and immune checkpoints were measured by qPCR; (Biological icon credits: Bioicons – bladder icon (adapted by including a bladder carcinoma (BLCA)) by Servier https://smart.servier.com/ is licensed under CC‐BY 3.0 Unported https://creativecommons.org/licenses/by/3.0/).

To assess the response of different bladder carcinomas to chemotherapy tissue slices were harvested from cystectomies, cultured and treated with Gemcitabine/Cisplatin at concentrations of 5× and 10× IC50 (IC50 Gemcitabine/Cisplatin: 0.25 μm/7.5 μm) (for workflow, see Fig. 1).

Caspase‐3 immunohistochemistry was performed to determine chemotherapy‐induced apoptosis.

The mean caspase‐3‐expression in uncultured samples was 0.5 ± 0.005%. Cultivation of tissue slices for two days in the specialized medium led to an increase in caspase‐3‐expression to 6 ± 0.05%. After treatment with 5× and 10× IC50 Gemcitabine/Cisplatin, caspase‐3‐expression increased to 11 ± 1.2%. The immunohistochemical expression of the chemotherapy‐treated tissue slices was normalized to the expression of tissue cultivated in medium, resulting in a mean treated expression of 1190 ± 36.8%. When differentiating between Responders and Non‐Responders, significant differences were observed in the normalized caspase‐3 values. In the Responder group (*n* = 7), the mean uncultured caspase‐3‐expression was 115 ± 1.85%, increasing to 2578 ± 50.78% after 10× IC50 treatment and 2403 ± 41.78% when combining both treatment conditions. In contrast, Non‐Responders (*n* = 8) exhibited a markedly lower response, with an uncultured mean expression of 9 ± 0.12%, rising only to 119 ± 0.89% after 10× IC50 treatment and 140 ± 1.98% under both treatment conditions.

Ki67, a proliferation marker, was used to evaluate cellular proliferation under different conditions. The mean immunohistochemical Ki67‐expression in uncultured samples was 37 ± 0.21%. Under MSC culture conditions, Ki67 expression dropped to 13 ± 0.15%. After chemotherapy treatment, Ki67 levels were 14 ± 0.15%. When differentiating between Responders and Non‐Responders—based on the classification through caspase‐3‐expression—Responders showed a Ki67‐decrease from 9 ± 0.1% (untreated) to 8 ± 0.09% (treated), while Non‐Responders exhibited an increase from 16 ± 0.18% (untreated) to 21 ± 0.20% (treated). The data suggest that Ki67‐expression remained largely unchanged between untreated and chemotherapy‐treated samples, indicating that the treatment did not significantly reduce proliferation. Thus, Ki67‐expression alone does not appear to be a suitable marker for categorizing Responders and Non‐Responders. However, the presence of Ki67 staining confirms that the tissue remained viable throughout the culture and treatment process.

### Chemotherapy results in specific pathway downregulation

**Fig. 2:**
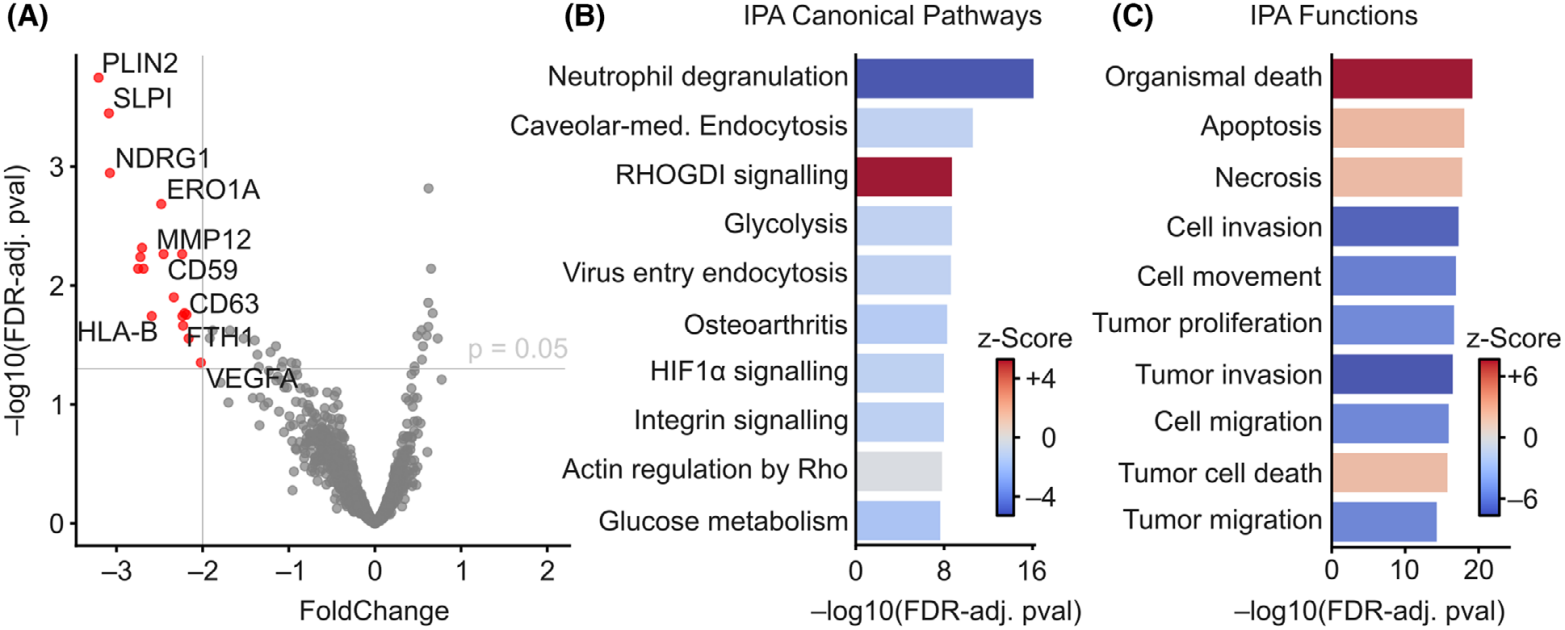
Response to gemcitabine/cisplatin treatment. (A) Vulcano plot of 18 677 transcripts showing significant transcripts in tumor regions after treatment with Gem/Cis compared to adjacent medium‐treated tumor regions. FDR‐adjusted p‐values below 0.05 and absolute FoldChange (FC) above 2 were deemed significant (red). (B, C) Ingenuity Pathway Analysis (IPA) of significant transcripts showing up‐ (red) and down‐regulated (blue) pathways of top ten significant pathways from the “Canonical Pathways” and “Functions” IPA libraries.

Treatment of tissue slices with Gem/Cis resulted in the downregulation of 18 transcripts (Fig. 2A). Ingenuity Pathway Analysis (IPA) of downregulated transcripts highlighted inactivation of processes that mediate substance uptake in cells (e.g., endocytosis). Also, metabolic processes (e.g., glycolysis and glucose metabolism) were downregulated after chemotherapy (Fig. 2B). Inhibition of Rho activities provides a possible link between integrin signaling and endocytotic processes, both requiring functional actin dynamics. When looking further into broader functional categories, cell death by apoptosis and necrosis were significantly enhanced, as can be expected after cytotoxic treatment (Fig. 2C). Motility and proliferation of cancer cells were also reduced after treatment.

### Resistance to chemotherapy is associated with stromal M2 macrophage recruitment

**Fig. 3:**
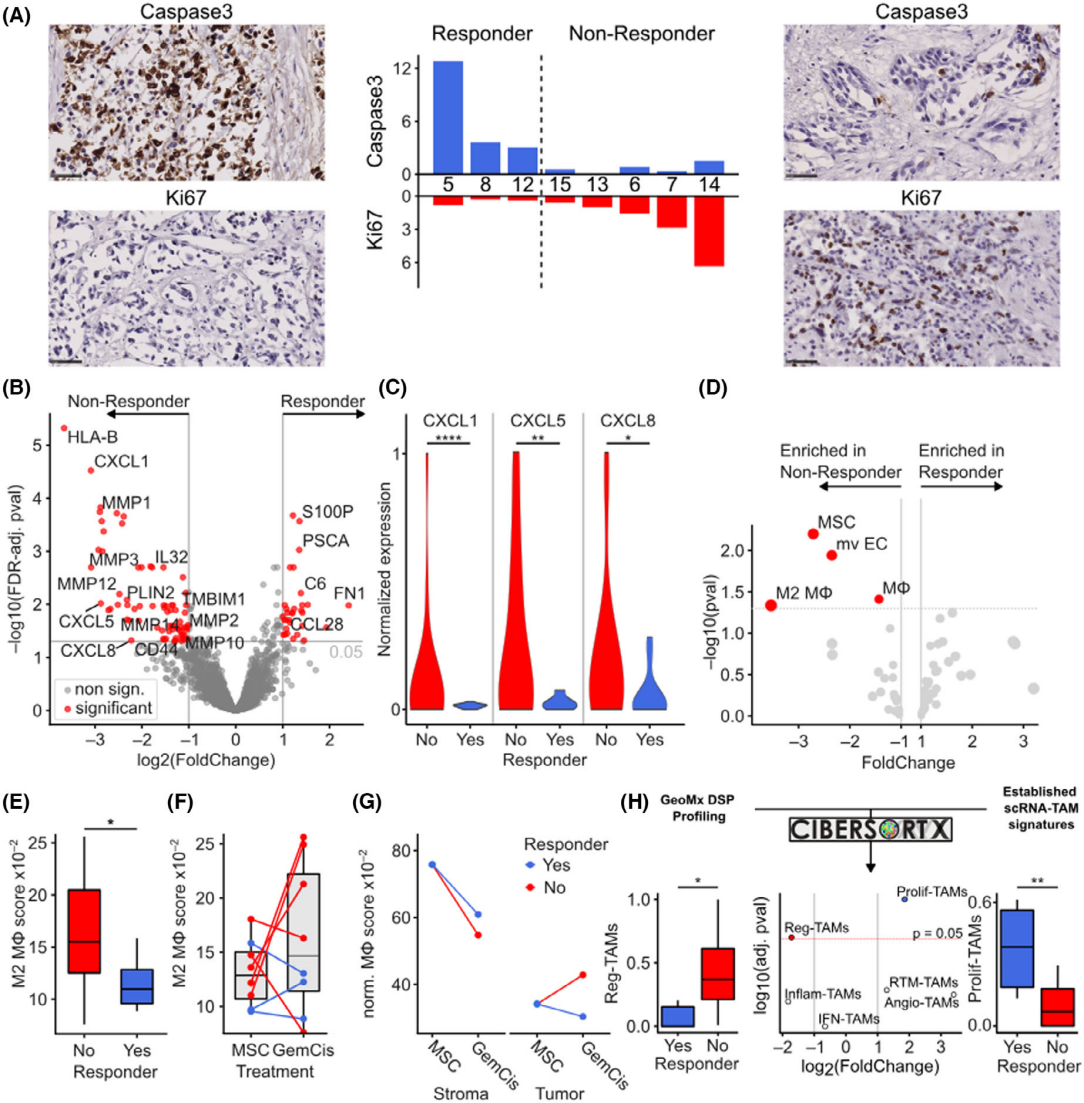
Differences between chemotherapy‐susceptible and resistant tumors. (A) Gem/Cis treated sections were stained with antibodies targeted against Caspase3 or Ki67. FoldChange of positive cells compared to medium‐treated adjacent control slides are displayed in the bar chart. Cutoff of Caspase3 FoldChange above 2.0 was used to differentiate susceptible (“Responder”) from resistant (“Non‐Responder”) tumors. (B) Vulcano plot of 18 677 transcripts showing significant transcripts in tumor regions between Responder and Non‐Responder tumor specimens after Gem/Cis treatment. *P*‐value below 0.05 and absolute FoldChange above 2 was deemed significant. (C) Violin plots of min‐max normalized expression values of chemokine transcripts within tumor regions of Gem/Cis treated samples. (D) Vulcano plot of main immune cell subpopulation enrichment scores compared between Non‐Responder (enriched on left) and Responder (enriched on right). Mφ = macrophage, MSC = mesenchymal stem cells, mv EC = microvascular endothelial cells. (E) Boxplot comparing M2 macrophage enrichment between Responder and Non‐Responder tumor spots (*n* = 3 Responder, *n* = 5 Non‐Responder, Mann Whitney *U* test). (F) Paired boxplot of Gem/Cis tumor spots with adjacent samples of medium‐treated controls (MSC) between Non‐Responder (red) and Responder (blue) classified patients (*n* = 3 Responder, *n* = 5 Non‐Responder). (G) Comparison of normalized macrophage enrichment between medium‐treated controls (MSC) and adjacent Gem/Cis treated samples for Stroma and Tumor ROIs respectively (*n* = 3 Responder, *n* = 5 Non‐Responder). (H) Subtyping of tumor‐associated macrophages (TAMs) into interferon‐primed TAMs (IFN‐TAMs), immune regulatory TAMs (Reg‐TAMs), inflammatory cytokine‐enriched TAMs (Inflam‐TAMs), lipid‐associated TAMs (LA‐TAMs), proangiogenic TAMs (Angio‐TAMs), resident‐tissue macrophage‐like TAMs (RTM‐TAMs) and proliferating TAMs (Prolif‐TAMs, *n* = 3 Responder, *n* = 5 Non‐Responder, FDR‐adj. Mann–Whitney U test). *P < 0.05, **P < 0.01, ****P < 0.0001.

Resistance to chemotherapy presents a significant challenge in cancer treatment. To investigate potential mechanisms underlying tumor resistance, patient samples were categorized into Responders and Non‐Responders based on Caspase‐3 expression, a protease upregulated during apoptotic cell death (Fig. 3A). Three samples exhibiting high Caspase‐3 expression were classified as Responders, while the remaining five samples were designated as Non‐Responders. Consistent with this classification, Ki67 protein expression, a marker of cellular proliferation, correlated with response status. Differential gene expression analysis between tumor regions of Responders and Non‐Responders following Gem/Cis treatment revealed upregulation of cytokines (e.g., *IL‐24, IL‐33*), chemokines (e.g., *CXCL1, CXCL5, CXCL8*), and extracellular matrix‐modifying enzymes (e.g., MMP1, MMP3) in Non‐Responders (Fig. 3B). In addition, *SPP1* was found to be significantly upregulated (FC = 2.0645; *P* = 0.0139) in Non‐Responder cells. In contrast, Responders exhibited increased expression of *CCL28* and components of the complement cascade (e.g., C6). Given the elevated chemokine expression in Non‐Responders (Fig. 3C), we investigated potential cellular targets of these chemoattractants. Spatial transcriptomic deconvolution enabled the identification and quantification of immune cell populations within tumor samples (Fig. 3D). Notably, Non‐Responder tumors exhibited significantly higher enrichment of mesenchymal stem cells (MSC), microvascular endothelial cells (mvEC), and M2 macrophages compared to Responders. M2 macrophages are well‐established promoters of tumor progression and chemoresistance, particularly in the context of gemcitabine and cisplatin therapy. The increased enrichment of M2 macrophages in Non‐Responders further supports their role in mediating resistance (Fig. 3E). Given the concurrent upregulation of macrophage‐recruiting chemokines and M2 macrophage enrichment in Non‐Responders, we hypothesized that intratumoral macrophage accumulation would be increased in Non‐Responders relative to medium‐treated controls (MSC). Indeed, Non‐Responders displayed a significant increase in M2 macrophage enrichment compared to controls (Fig. 3F). Utilizing spatial transcriptomics of tumor‐associated stromal regions, we observed that macrophage enrichment decreased in the stromal compartment of both Responders and Non‐Responders following treatment, with a more pronounced reduction in Non‐Responders (Fig. 3G). At the same time, macrophages increased within the tumor regions of Non‐Responders and decreased in Responders. In the tumoral compartment, macrophage enrichment and polarization increased in Non‐Responders compared to Responders following treatment, suggesting that Non‐Responders actively recruit macrophages from the tumor‐associated stroma and induce an M2 phenotype to evade the cytotoxic effects of Gem/Cis therapy. Subtyping of tumor‐associated macrophages (TAMs) revealed distinct polarization patterns between treatment response groups (Fig. 3H). Non‐Responders exhibited significant enrichment of immune regulatory TAMs (Reg‐TAMs), a subset characterized by extensive immunosuppressive properties [29]. Conversely, Responders demonstrated predominant enrichment of proliferating TAMs (Prolif‐TAMs), distinguished by their rapid proliferation kinetics and proinflammatory phenotype. This subset has been implicated in generating downstream TAM populations with enhanced antitumor cytotoxic capacity [29].

### Changes in tumor–stroma signaling as a result of Gem/Cis treatment

**Fig. 4:**
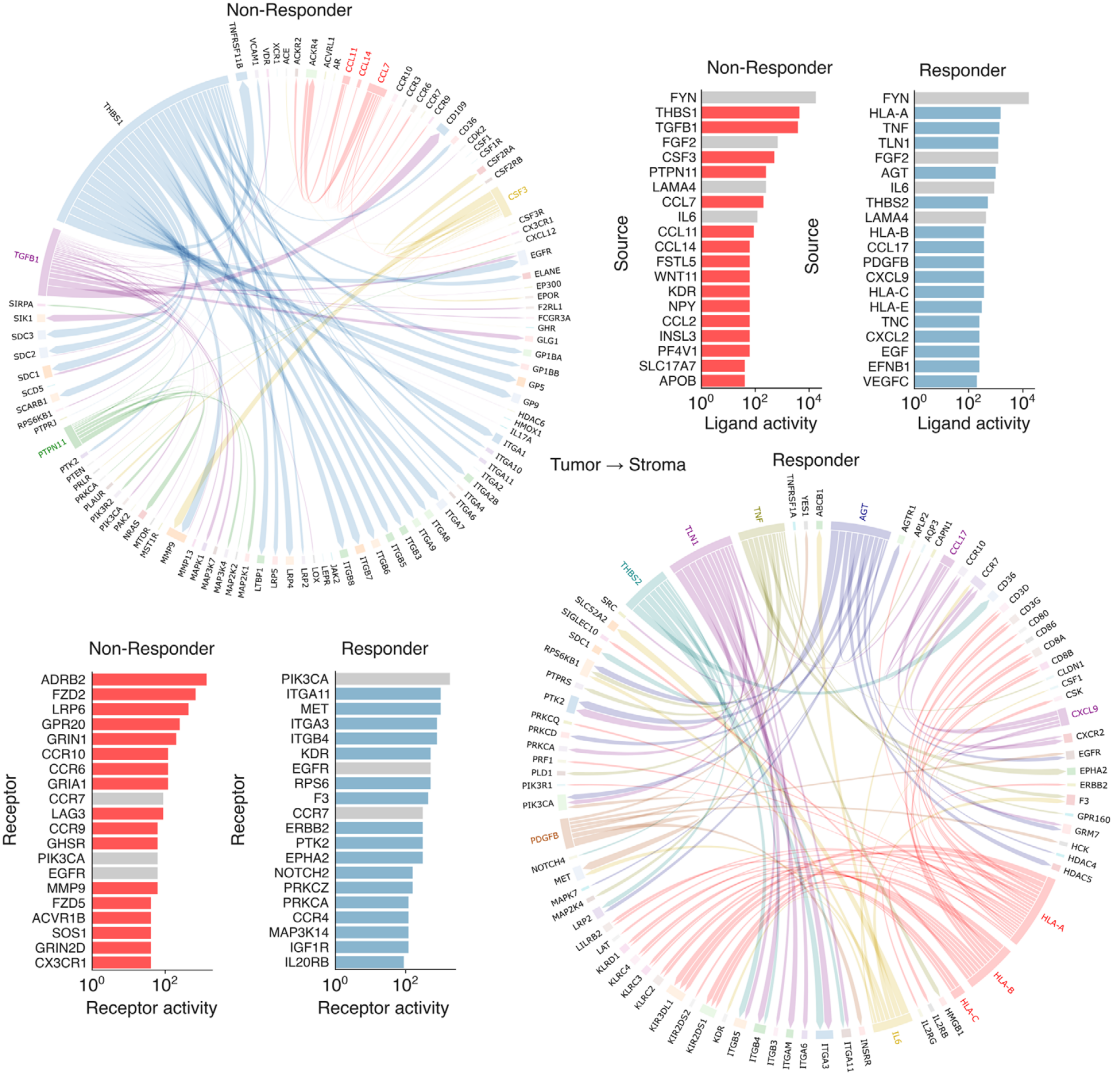
Ligand‐receptor interactions in chemotherapy susceptible and resistant tumors. Top ligands based on ligand activity signaling from stroma to tumor ROIs. Pearson correlation between a ligand and its targets was used to determine a ligand and receptor activities between two regions. Top left shows Non‐Responder after Gem/Cis treatment, while lower right shows Responders.

Having demonstrated that differential recruitment of immune cells from adjacent stromal regions plays a crucial role in therapy evasion, we sought to further investigate the interactions between tumor and surrounding stromal regions by mapping ligand–receptor signaling networks. In Non‐Responders, key chemokines involved in macrophage recruitment, specifically CCL7 and CCL14, activated stromal cells via their respective receptors (Fig. 4). CXCL1, CXCL5, and CXCL8 showed binding to respective receptors in Non‐Responder patients (Fig. S2). Tumor‐derived THBS1 (thrombospondin‐1) activated a variety of stromal integrins, which are critically involved in cell adhesion, migration, angiogenesis, and tumor progression [28]. Additionally, stromal matrix metalloproteinases (MMPs) were activated by CSF3, THBS1, and TGF‐β1. In contrast, Responders exhibited elevated signaling between tumor‐derived HLA molecules and stromal immune‐activating molecules (e.g., CD80, CD86). Furthermore, tumor‐derived chemokines such as CXCL9, binding to its respective stromal receptors, were shown to recruit cytotoxic T cells, Th1 cells, and NK cells, all of which possess potent antitumoral activity [30].

### Functional validation of overexpressed immunomodulating markers in Non‐Responder compared to Responder cancer cells

**Fig. 5:**
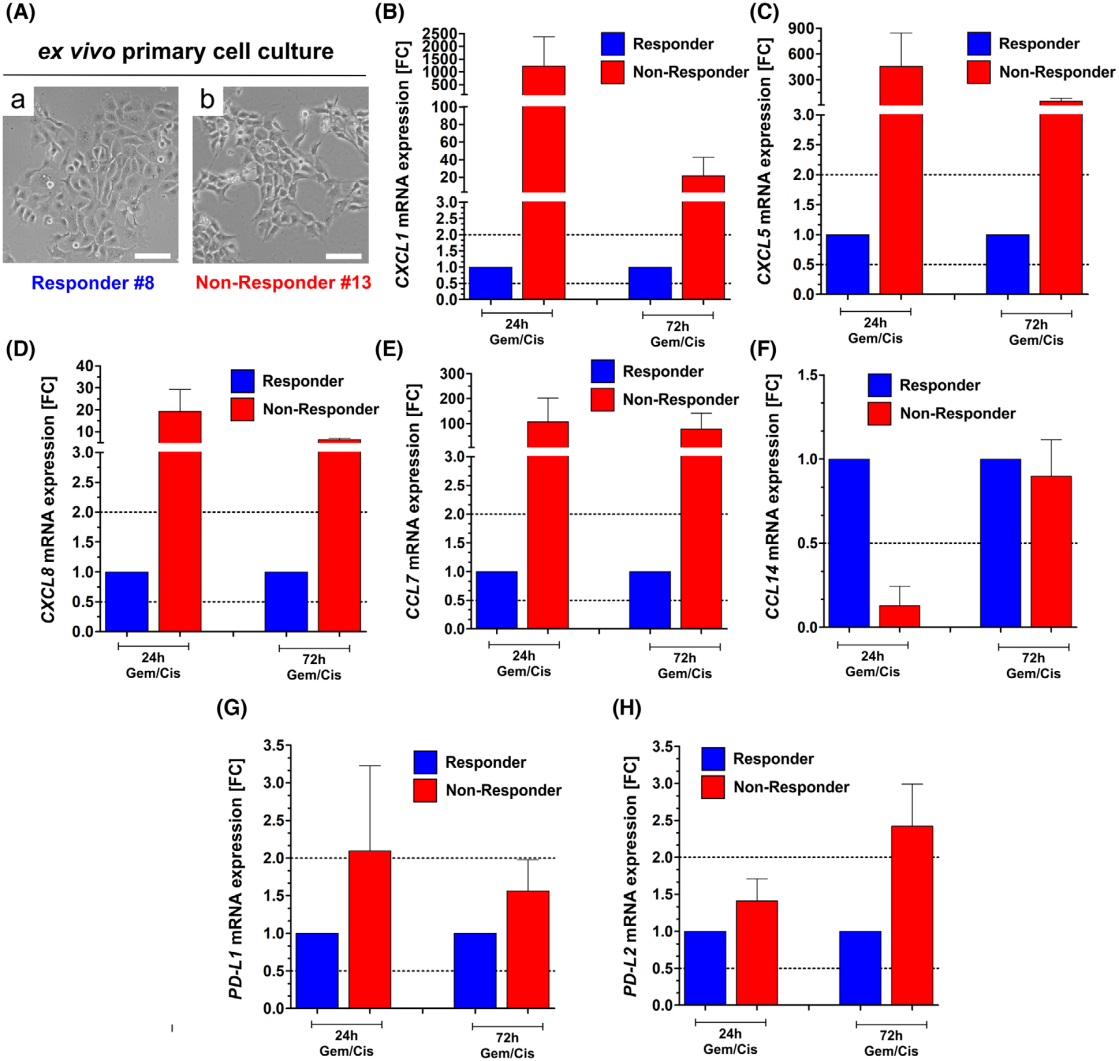
Functional validation of overexpressed immunomodulating markers in Non‐Responder compared to Responder cancer cells. (A) Representative images of primary cancer cell cultures established from tissue slice corresponding tissue samples (Responder and Non‐Responder); scale bar: 100 μm. (B–H) Relative mRNA expression of cytokines (*CXCL1, CXCL5, CXCL8, CCL7, CCL14*) and immune checkpoint genes (*PD‐L1* and *PD‐L2*) was determined 24 and 72 h after Gem/Cis treatment of Responder and Non‐Responder cancer cells. Mean values with SEM are shown (*n* = 2 independent experiments).

In order to confirm overexpression of chemokines as identified by transcriptomic analysis, primary cancer cell cultures were established from tissue slice corresponding tissue samples (Responder and Non‐Responder, see Fig. 1 and Fig. 5A) and treated with Gem/Cis (0.1 μm/1.0 μm) for 24 and 72 h *in vitro*. qPCR analyses verified increased expression levels of *CXCL1*, *CXCL5* and *CXCL8* (Fig. 5B–D) in Non‐Responder cell cultures at both end points. *CXCL1* mRNA was upregulated up to 1220 fold compared to the Responder cancer cells. Overexpression of *CCL7* was also confirmed by qPCR while *CCL14* did not show any differential expression between Responder and Non‐Responder cells upon Gem/Cis treatment (Fig. 5E,F). Since cancer cell‐specific upregulation of chemokines might be involved in recruitment of immune cells such as cytotoxic T cells (beyond macrophages), we finally determined mRNA levels of the immune checkpoints *PD‐L1* and *PD‐L2* as putative protective mechanism to evade counteracting effects. At this, we observed in Non‐Responder cancer cells a slight upregulation of both *PD‐L1* and *PD‐L2* mRNA expression compared to Responder cells (Fig. 5G,H).

### *In silico* validation of Non‐responder gene signature using publicly available TCGA data

**Fig. 6:**
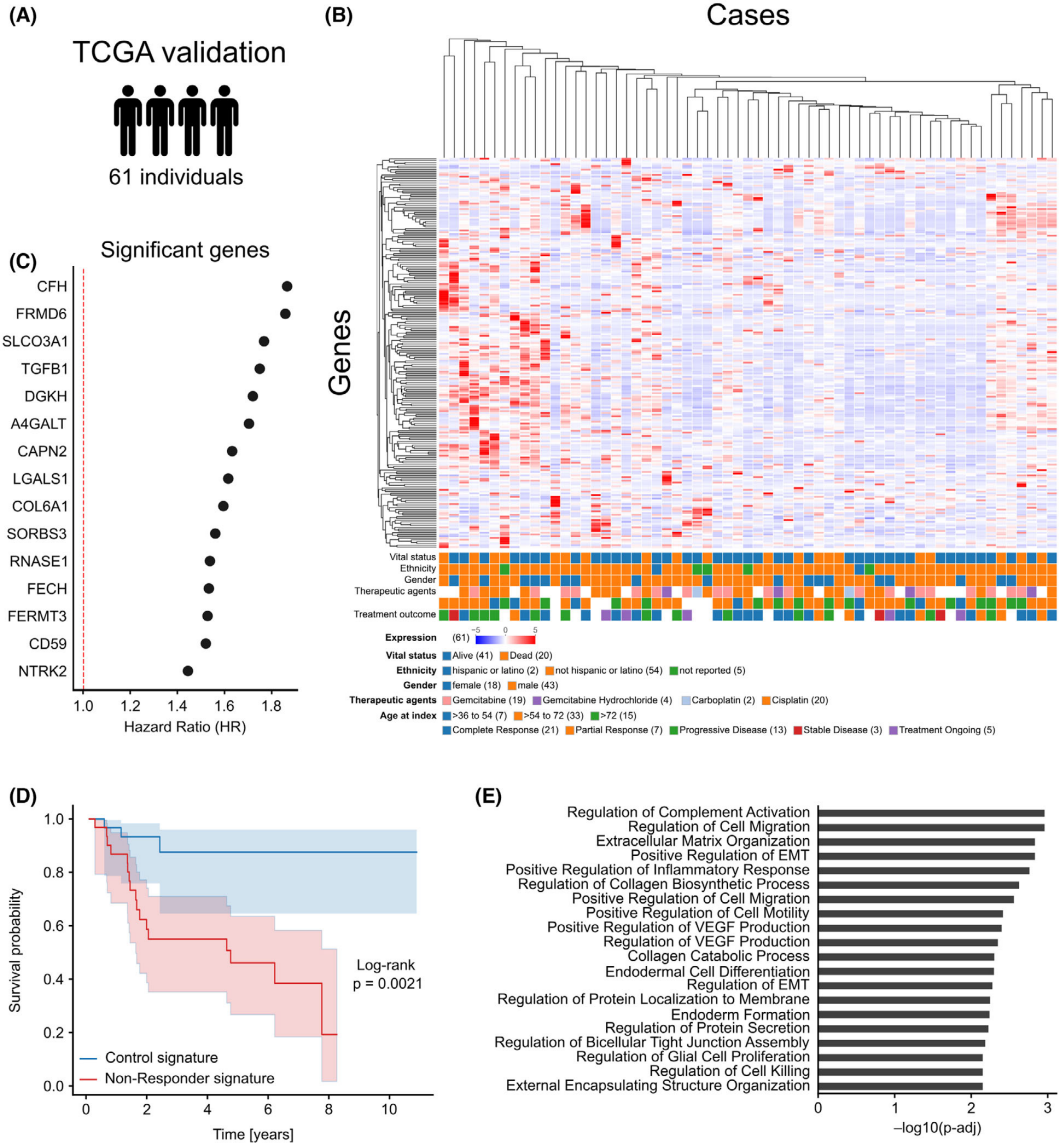
*In silico* validation of Non‐Responder gene signature using publicly available TCGA data. (A) TCGA bladder cancer cohort of patients treated with platinum‐based and gemcitabine‐containing regimens (*n* = 61). (B) Unsupervised hierarchical clustering heatmap of previously identified Non‐Responder signature genes. (C) Hazard ratio analysis showing genes from the Non‐Responder signature associated with increased mortality risk. (D) Kaplan–Meier survival curves demonstrating significantly worse overall survival in patients with upregulated Non‐Responder signature genes (log‐rank test). (E) Gene ontology pathway enrichment analysis of Non‐Responder signature genes with significant (*P* < 0.05) hazard ratios.

To validate the previously established transcriptomic signature of Non‐Responder cancer cells, publicly available RNA‐seq data from The Cancer Genome Atlas (TCGA) were analyzed. We identified 61 patients who received platinum‐based (cisplatin or carboplatin) and gemcitabine‐containing chemotherapy regimens (Fig. 6A). Expression data for all transcripts previously found to be upregulated in Non‐Responders were available in these cases (Fig. 6B). All transcripts that showed a significant hazard ratio demonstrated positive (unfavorable) associations with survival (Fig. 6C), consistent with the Non‐Responder phenotype observed in our cohort. Patients with high expression of this composite transcriptomic signature exhibited significantly worse overall survival (*P* = 0.0021) in Kaplan–Meier analysis (Fig. 6D). Pathway enrichment analysis using the GO Biological Process 2025 database revealed pathways previously associated with treatment resistance (Fig. 6E). Notably, cell migration pathways were enriched, consistent with the recruitment of M2 macrophages reported earlier. Stromal interaction pathways were also significantly enriched, including processes related to angiogenesis (e.g., VEGF production) and extracellular matrix remodeling. Together, these findings validate the Non‐Responder gene signature identified in our experimental system.

## Discussion

Despite limited response rates, cisplatin‐based chemotherapy is the key substance in perioperative systemic therapy of muscle‐invasive bladder cancer and remains a component in the treatment of advanced bladder cancer. Increasing evidence suggests that molecular subtypes and genomic alterations may influence cisplatin sensitivity in this heterogeneous disease [31]. However, the role of the tumor microenvironment—particularly stromal components—in modulating chemotherapy response remains incompletely understood. In this study, we employed an *ex vivo* tissue slice model to investigate for the first time differential molecular signatures in both tumor and stromal compartments of bladder cancer upon treatment. By integrating spatially resolved transcriptomic profiling, we aimed to identify microenvironmental features that may contribute to chemoresistance in bladder cancer and identify expression patterns potentially associated with chemotherapy response.

Spatial transcriptomic analysis revealed distinct patterns of gene expression in the tumor and stromal compartments that differed markedly between Responders and Non‐Responders. The immunomodulating gene *SPP1* has been identified to be upregulated [32]. Notably, Sjödahl *et al*. proposed SPP1 as a putative predictive biomarker whose overexpression was associated with lower response rates to neoadjuvant chemotherapy in bladder cancer of the Ba/Sq subtype [33]. Beyond that, Non‐Responders exhibited a significant upregulation of chemokines such as *CXCL1*, *CXCL5*, and *CXCL8*, which were confirmed by primary cell cultures upon Gem/Cis treatment *in vitro*. All of which have been previously implicated in promoting tumor progression, angiogenesis, and treatment resistance. CXCL5, for instance, has been shown to enhance tumor invasiveness via the PI3K/AKT‐mediated upregulation of MMP2 and MMP9, while also contributing to chemoresistance through NF‐κB activation [34, 35]. Similarly, increased CXCL1 expression has been associated with poor survival and advanced grade and stage in bladder cancer [36]. CXCL1 was also found to promote the recruitment of tumor‐associated macrophages (TAMs) and cancer‐associated fibroblasts (CAFs), reinforcing stromal–tumor interactions that contribute to immune suppression and relapse [37]. These findings suggest that the upregulation of CXCL chemokines may serve as a critical mechanism underlying stromal–tumor crosstalk and chemoresistance.

TAMs represent a heterogeneous and plastic population of immune cells within the tumor microenvironment. They can adopt either a pro‐inflammatory, tumoricidal M1 phenotype or an immunosuppressive, tumor‐promoting M2 phenotype, depending on local cues. M1 macrophages are classically activated by microbial products and Th1 cytokines such as IFN‐γ, and are characterized by the production of reactive oxygen and nitrogen species as well as inflammatory cytokines. In contrast, M2 polarization is driven by anti‐inflammatory signals including IL‐4, IL‐10, and IL‐13, and results in macrophages that promote tissue remodeling, angiogenesis, and immune evasion [38]. A growing body of evidence suggests that TAMs in many solid tumors predominantly adopt an M2‐like phenotype, which has been linked to immune evasion and poor clinical outcomes. Animal models have shown an early increase of M2 macrophages during tumorigenesis in multiple tumor types including lung and prostate [39]. Moreover, in bladder cancer, the presence of M2 macrophages has been associated with an immunosuppressive tumor environment and a more aggressive phenotype. For example, Lima et al. demonstrated increased expression of CD163‐positive M2 macrophages in patients with squamous cell carcinoma of the bladder, alongside upregulation of immune checkpoint molecules such as PD‐1 and CTLA‐4 [40].

In line with these findings, our data show that chemotherapy Non‐Responders displayed a significant enrichment of M2 macrophages within the tumor compartment, alongside a depletion of macrophages in the adjacent stromal regions. This spatial shift suggests active recruitment of stromal macrophages into the tumor microenvironment, where they likely undergo M2 polarization. The concurrent upregulation of M2‐polarizing cytokines and chemokines such as CXCL1 supports a model in which chemotherapeutic pressure may induce or amplify an immunosuppressive niche, fostering tumor persistence. At this, the CXCL8‐CXCR1/2 axis may play a crucial role in TAM mobilization and recruitment [41] suggesting a rationale for our observed enrichment of M2 macrophages in tumor areas. In contrast, chemotherapy Responders that exhibited reduced CXCL8 expression are characterized by a reduction of M2 macrophages in both compartments, indicating a fundamentally different microenvironmental response to treatment. This observation was further strengthened by TAM subtyping, which revealed the presence of immunoregulatory Reg‐TAMs in Non‐Responders opposed to proinflammatory Prolif‐TAMs in Responders.

Given the prominent role of M2‐polarized TAMs in chemoresistant tumors, strategies targeting these cells represent a viable strategy for therapeutic intervention. Approaches currently under investigation include inhibition of monocyte recruitment (e.g., CCL2‐CCR2 blockade) [42, 43] and macrophage depletion via CSF‐1/CSF‐1R inhibition. Notably, CSF‐1R inhibition has been shown to reduce TAM density and enhance CD8+ T cell infiltration, although compensatory mechanisms may limit long‐term efficacy [44]. An alternative strategy involves macrophage re‐education toward the antitumoral M1 phenotype using agents such as IFN‐γ, CD40 agonists, or PI3Kγ inhibitors, which can shift TAMs toward an immune‐stimulatory state and enhance antitumor immunity [45, 46]. In addition, inhibitors targeting the CXCL8‐CXCR1/2 axis are thought to be promising approaches to diseases involving immune cell crosstalk including cancer therapy. Compounds such as Reparixin are currently tested in clinical phase I and II trials [47]. Already in 2010, Ginestier et al. demonstrated reduced cancer (stem) cell growth in breast cancer patient‐derived xenograft models when combining Reparixin with chemotherapeutics (docetaxel) [48]. Dabkeviciene et al. showed that CXCL8 is upregulated in a chemoresistant subline of colorectal cancer cells whereas modulation of the CXCR2 pathway suppresses the growth of chemoresistant cancer cells [49]. Beyond that, there are accumulating studies providing evidence that CXCR2 blockade correlates with favorable outcomes in various tumor entities including pancreatic [50] and prostate cancer [51].

Furthermore, immune checkpoint blockade with anti‐PD‐1/PD‐L1 antibodies has been demonstrated to promote macrophage maturation and shift the TAM population toward a pro‐inflammatory phenotype by promoting the production of IFN‐γ in CD8+ T cells [52]. These findings suggest that combining chemotherapy with TAM‐targeted or immunomodulatory therapies may help overcome stromal‐mediated resistance mechanisms in bladder cancer and promote the addition of perioperative immunotherapy in these patients [53] while highlighting for the first time the involvement of TAMs in the chemotherapy response of bladder cancer. This notion was supported by our *in vitro* cultured primary cancer cells that revealed a clear trend for upregulation of *PD‐L1* and *PD‐L2* in Non‐Responder cancer cells.

However, our study holds a few limitations since an active recruitment of macrophages due to the expression of chemokines such as CXCL8 remains speculative. In addition, the microenvironmental interplay of the immunomodulated markers and factors including those of the complement system is known to be complex, varying between different tumor entities and has been described as having opposing effects, that is, tumor‐promoting or tumor‐suppressive [54, 55, 56]. For instance, associations of CCL28 expression, upregulated in Responders, with clinical and prognostic outcomes have been controversially described in the context of breast cancer subtypes [57]. The same for the factors of the complement system such as C6 (also upregulated in Responder tissues), that is, various Fc chain receptors and CD59 whose overexpression correlated in some cancers with poor prognosis while others showed favorable outcomes [58, 59]. This might be explained by the ambivalent nature of the complement cascade, which either mediates antitumoral effects via opsonophagocytosis and cytotoxicity or exerts a C5a/C5aR1‐driven and pro‐tumoral influence on chronic inflammation [60]. Thus, simple interventions based on novel therapeutic concepts such as inhibitors of the immune cell crosstalk appear to be challenging and require further in‐depth research to decipher the exact network and interplay of identified markers and their impact on chemotherapeutic response rates in bladder cancer.

## Conflict of interest

The authors declare no conflict of interest.

## Author contributions

MR, NTG, and SL were involved in conception and design. SL harvested tissues, cultured, treated, and immunohistochemically stained patient‐derived tissues slices. JR and LS performed/supported data analyses of spatial transcriptomics. MS was involved in providing patient samples. JP, CF, and CM supported *ex vivo* culturing/treatment and generation of tissue slices. MR, NTG, PB, and DJ discussed/supervised the project. SL, JR and MR wrote the original draft. All authors edited, critically read, reviewed, and agreed to the submitted version of the manuscript.

## Supporting information


**Fig. S1.** Schematic overview of tumor‐stroma segmentation.

**Fig. S2.** Ligand–receptor interactions of relevant chemokines from tumor to stroma regions in Non‐Responder patients involving CXCL1, CXCL5 and CXCL8.

**Table S1.** Cohort characteristics of *ex vivo* treated bladder cancers.

**Table S2.** IC50 values as previously published (Rose et al. 2020).

**Table S3.** Primer sequences and PCR conditions.

## Data Availability

The data that support the findings of this study are openly available in genomics data repository Gene Expression Omnibus (GEO accession: GSE296955; reviewer access token: gnuzqeyovfsbnod).
