# Supplementary material for: Tumor‐stromal crosstalk and macrophage enrichment are associated with chemotherapy response in bladder cancer
Source: FEBS Open Bio. 2025 Dec 12;16(6):1197–212. doi: 10.1002/2211-5463.70179 (PMC13238752; doi:10.1002/2211-5463.70179)
Supplement: Supplementary file 1 — Fig. S1. Schematic overview of tumor‐stroma segmentation. [file FEB4-16-1197-s003.docx]

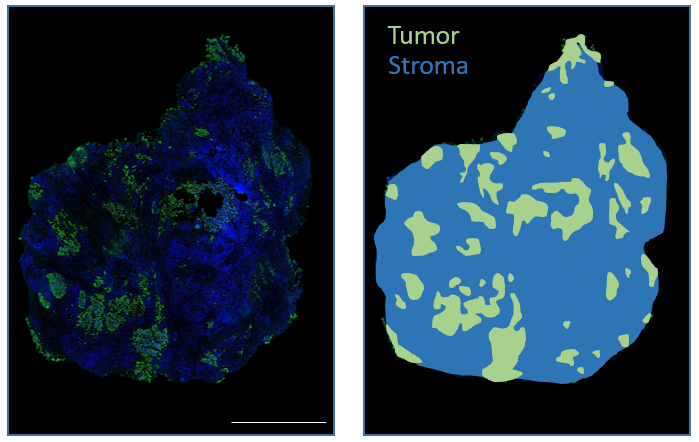


**Supplemental Figure 1. Schematic overview of tumor-stroma segmentation.**Tissue sections were formalin-fixed, paraffin-embedded, and stained with Syto13 (nuclei, blue) and PanCK (tumor, green), as shown on the left. A representative schematic of manual whole-slice segmentation is shown on the right for illustrative purposes. Tumor regions were identified based on PanCK positivity; surrounding tissue was designated as stroma.
