# Supplementary material for: Tumor‐stromal crosstalk and macrophage enrichment are associated with chemotherapy response in bladder cancer
Source: FEBS Open Bio. 2025 Dec 12;16(6):1197–212. doi: 10.1002/2211-5463.70179 (PMC13238752; doi:10.1002/2211-5463.70179)
Supplement: Supplementary file 2 — Fig. S2. Ligand–receptor interactions of relevant chemokines from tumor to stroma regions in Non‐Responder patients involving CXCL1, CXCL5 and CXCL8. [file FEB4-16-1197-s005.docx]

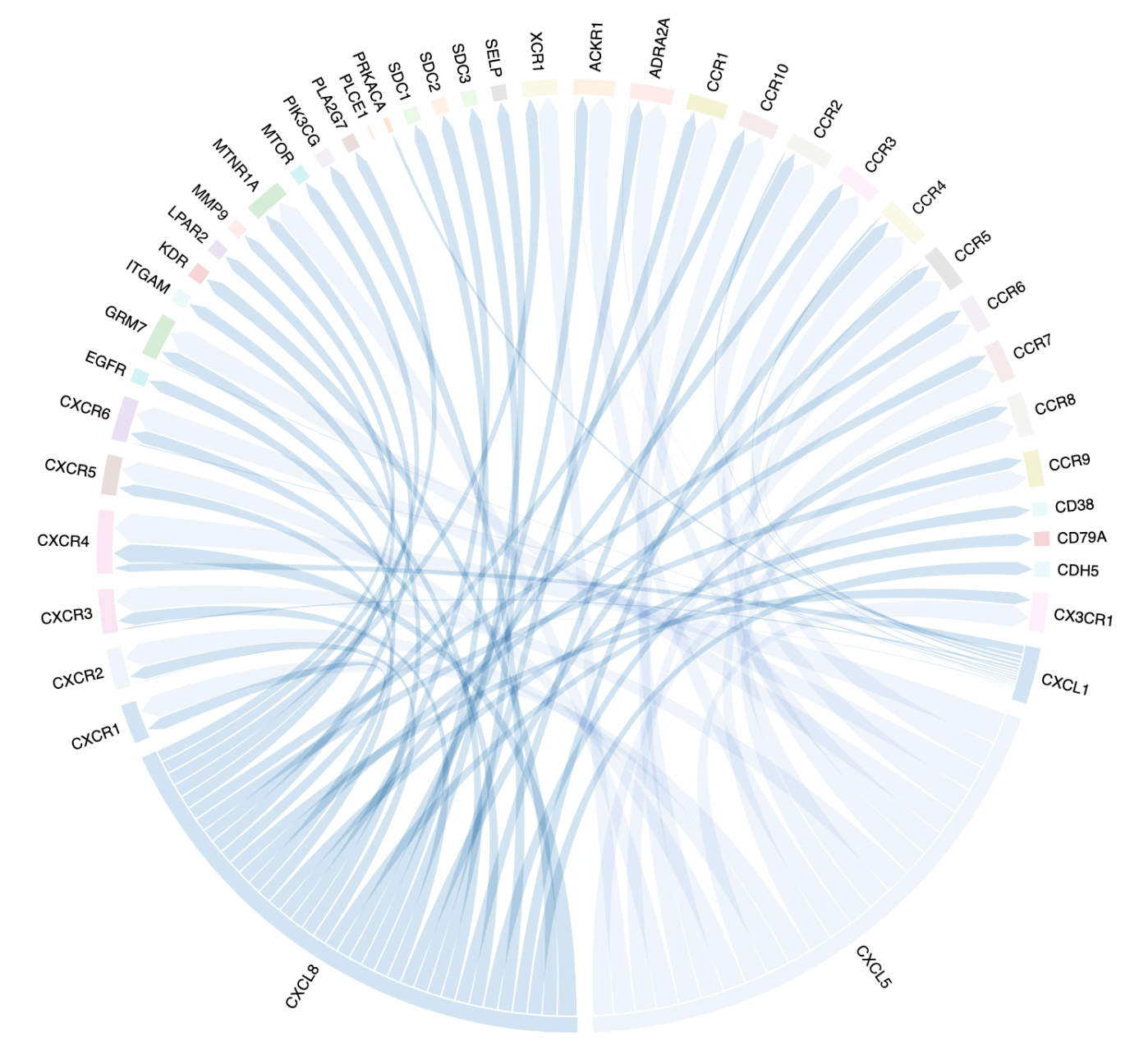


**Supplemental Figure 2: Ligand-receptor interactions of relevant chemokines from tumor to stroma regions in Non-Responder patients involving CXCL1, CXCL5 and CXCL8.** Pearson correlations between ligand and receptor targets were used to determine ligand-receptor activities.
