# Supplementary material for: Tumor‐stromal crosstalk and macrophage enrichment are associated with chemotherapy response in bladder cancer
Source: FEBS Open Bio. 2025 Dec 12;16(6):1197–212. doi: 10.1002/2211-5463.70179 (PMC13238752; doi:10.1002/2211-5463.70179)
Supplement: Supplementary file 3 — Table S1. Cohort characteristics of ex vivo treated bladder cancers. [file FEB4-16-1197-s002.docx]

**Supplemental Table 1: cohort characteristics of ex vivo treated bladder cancers**

|  | Total cohort  n = 15 |
| --- | --- |
| Age* [years] | 71 ± 5 |
| Gender   - Male - Female | 10  5 |
| Entity   - Urothelial carcinoma - Urothelial carcinoma with divergent differentiation - Squamous cell carcinoma | 7 4  4 |
| Grading   - G1 - G2 - G3 - G4 | 0 0 15 0 |
| Pathological T stage   - pT1 - pT2a - pT2b - pT3a - pT3b - pT4a - pT4b | 0 0 0 5 6 4 0 |
| Pathological N stage   - pN0 - pN1 - pN2 - pN3 | 9 2 2 2 |
| Lymph angioinvasion   - L0 - L1 | 9 6 |
| Venous angioinvasion   - V0 - V1 | 9 6 |
| Perineural growth   - Pn0 - Pn1 | 12 3 |
| Neoadjuvant Chemotherapy   - Yes - No | 2 13 |

* Median ± interquartile range (IQR)
