# Supplementary material for: Tumor‐stromal crosstalk and macrophage enrichment are associated with chemotherapy response in bladder cancer
Source: FEBS Open Bio. 2025 Dec 12;16(6):1197–212. doi: 10.1002/2211-5463.70179 (PMC13238752; doi:10.1002/2211-5463.70179)
Supplement: Supplementary file 4 — Table S2. IC50 values as previously published (Rose et al. 2020). [file FEB4-16-1197-s004.docx]

| **Supplemental Table 2: IC50 values as previously published (**Rose et al. 2020 [20]**)** | | |
| --- | --- | --- |
|  |  |  |
| **Cell line** | **IC50 value Gemcitabine [µM]** | **IC50 value Cisplatin [µM]** |
|  |  |  |
| **SCaBER** | 0.5778 | 10.71 |
| **HT1376** | 0.6464 | 12.03 |
| **J82** | 0.00896 | 5.011 |
| **RT112** | 0.009315 | 31.1 |
