# Supplementary material for: Tumor‐stromal crosstalk and macrophage enrichment are associated with chemotherapy response in bladder cancer
Source: FEBS Open Bio. 2025 Dec 12;16(6):1197–212. doi: 10.1002/2211-5463.70179 (PMC13238752; doi:10.1002/2211-5463.70179)
Supplement: Supplementary file 5 — Table S3. Primer sequences and PCR conditions. [file FEB4-16-1197-s001.docx]

**Supplementary Table 3:** Primer sequences and PCR conditions

**Supp. Table 3.1:** Primer sequences for RNA analyses

| Gene | Orientation | Primer sequences  5’ 🡪 3’ | Annealing temperature [°C] | Product size [bp] |  |
| --- | --- | --- | --- | --- | --- |
| *CCL14* | fwd  rev | CCT TAC CAC CCC TCA GAG TG  GGT GAT GAA GAC AAT TCC GGG | 63  61 | 20  21 |  |
| *CCL7* | fwd  rev | GGG CTG AGA CCA AAC CAG AA  TAA TCC CAA CTG GCT GAG CA | 60  58 | 20  20 |  |
| *CXCL1* | fwd  rev | GTC CGT GGC CAC TGA ACT  AGT GTG GCT ATG ACT TCG GT | 58  58 | 18  20 |  |
| *CXCL5* | fwd  rev | GTG TTG AGA GAG CTG CGT TG  GAG GCT ACC ACT TCC ACC TT | 60  60 | 20  20 |  |
| *CXCL8* | fwd  rev | GAG AGC TCT GTC TGG ACC C  TGA ATT CTC AGC CCT CTT CAA A | 62  58 | 19  22 |  |
| *PD-L1* | fwd  rev | GGT CAT CCC AGA ACT ACC TCT  TGA GTT TGT ATC TTG GAT GCC A | 60  60 | 21  22 |  |
| *PD-L2* | fwd  rev | CTT TGG CCA GCA TTG ACC TT  TTA GGG CTA TCA CTG TGG CT | 60  60 | 20  20 |  |
| *GAPDH* | fwd  rev | GAA GGT GAA GGT CGG AGT CA  AAT GAA GGG GTC ATT GAT GG | 60  60 | 20  20 |  |

**Supp. Table 3.2:** Mastermix for qPCR

| Substances | Volume [µl] |
| --- | --- |
| iQ5 SYBR-Green PCR Mix | 5 |
| Primer Mix:  10 µl Primer rev [10M]  10 µl Primer fwd [10M]  80 µl H_2_O | 0.5 |
| DNase/RNase free H_2_O | 3.5 |
| over all | **9** |

PCR-reaction volume of 10 µl over all, consisting of 9 µl Mastermix

and 1 µl cDNA.

**Supp. Table 3.3:** Cycle-Conditions of qPCR.

| Cycle | Repetitions | Time | Temperature [°C] |
| --- | --- | --- | --- |
| 1 | 1 | 30 sec | 95 |
| 2 | 40 | 5 sec | 95 |
| 3 | 40 | 30 sec | Tm |
| 4 | 1 | 5 sec | 65 |
| 5 | 1 | 0,5°C/cycle | 95 |
| 6 | 1 | hold | 4 |

Tm = annealing temperature.
